# Reply to: Fire activity as measured by burned area reveals weak effects of ENSO in China

**DOI:** 10.1038/s41467-022-32014-8

**Published:** 2022-07-28

**Authors:** Qichao Yao, Keyan Fang, Tinghai Ou, Feifei Zhou, Maosheng He, Ben Zheng, Jane Liu, Hang Xing, Valerie Trouet

**Affiliations:** 1 Wildfire Research Center, National Institute of Natural Harzards, 1 Anningzhuang Rd., Haidian, Beijng 100085 China; 2 Key Laboratory of Humid Subtropical Eco-geographical Process (Ministry of Education), College of Geographical Sciences, Fujian Normal University, Fuzhou, 350007 China; 3 Regional Climate Group, Department of Earth Sciences, University of Gothenburg, Gothenburg, 460 Sweden; 4 Department of Statistics, Colorado State University, Fort Collins, CO 80523 USA; 5 Department of Geography and Planning, University of Toronto, Toronto, ON 3359 Canada; 6 Laboratory of Tree-Ring Research, University of Arizona, Tucson, AZ 85721 USA

**Keywords:** Natural hazards, Fire ecology

**replying to** V. Resco de Dios et al. *Nature Communications* 10.1038/s41467-022-32013-9 (2022)

In their letter, Resco de Dios et al. discuss fire regimes in China using a dataset of annual area burned from a satellite product, i.e., the Moderate Resolution Imaging Spectroradiometer (MODIS) Collection 6 (C6) MCD 64 A1. They suggest that: (1) the fraction of burned area in China’s subtropics is low, (2) no dipole pattern exists between the burned area in western and eastern subtropical China, (3) the El Niño-Southern Oscillation (ENSO) has only weak effects on area burned in subtropical China, and (4) the fire suppression policy has little effect on fire activity in subtropical China. Their analysis of satellite-derived annual area burned raises some interesting issues that deserve further discussion.

## The satellite data overestimate wildfire activity in northern China

**Fig. 1:**
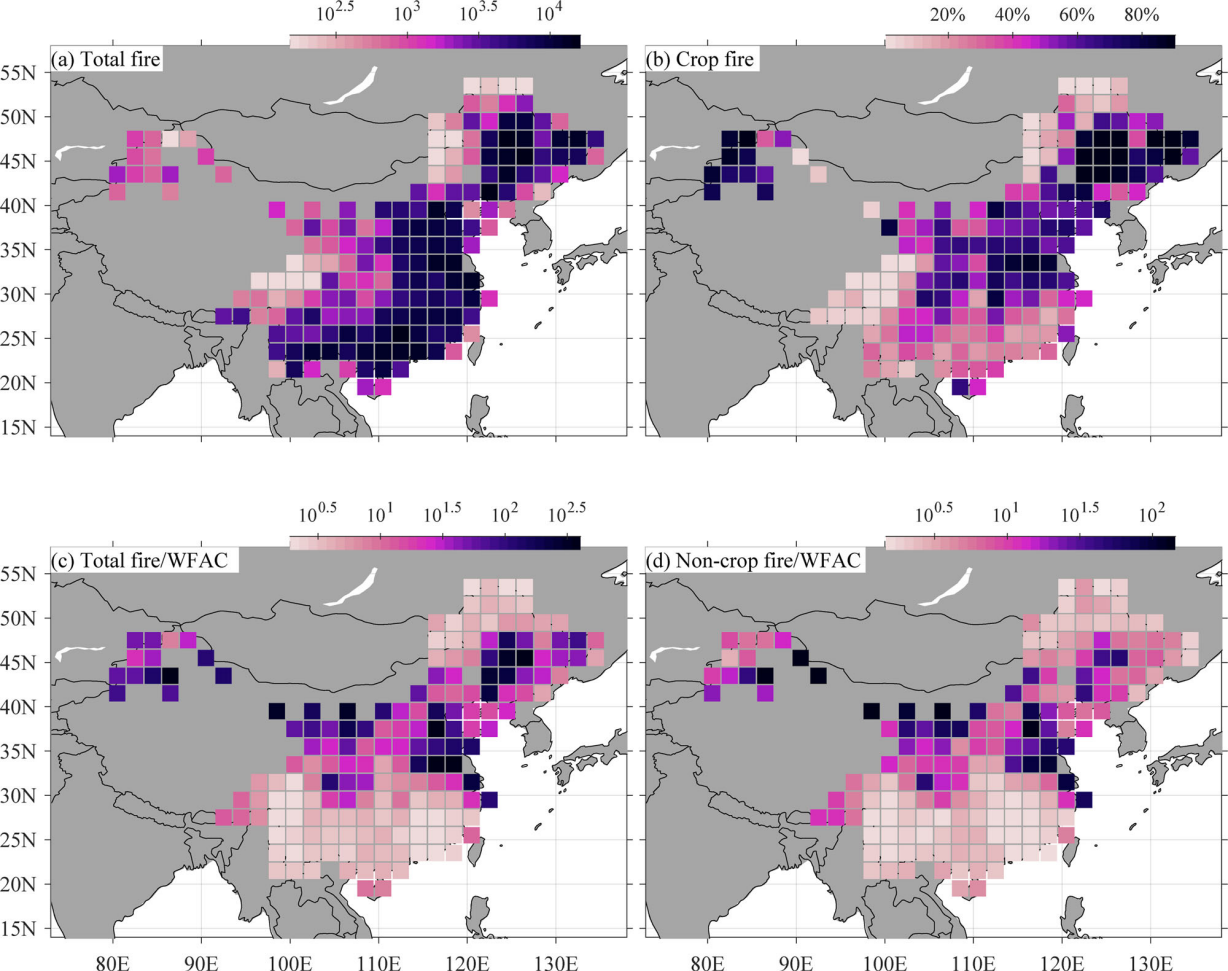
Fire occurrence comparisons between fire datasets. The fire occurrence numbers are from the Wildfire Atlas of China (WFAC) and the Moderate Resolution Imaging Spectroradiometer (MODIS) data, including MOD14A1 provided by Terra and MYD14A1 provided by Aqua^4^. **a** Total fire occurrences from MODIS, **b** crop fire occurrences from MODIS, **c** the ratio between total fire occurrences from MODIS and WFAC, and **d** the ratio between non-crop fire occurrences from MODIS and the WFAC. The fire occurrences were aggregated via combining the spatiotemporal adjacent fire points from MODIS Terra and Aqua from 2005 to 2018 into one fire event. Crop fire was picked out based on the land cover data of China in 2018^11^. The The 2 × 2 degree fire data is a sum of the fire occurrences.

The different wildfire data used in Fang et al. (2021) and Resco de Dios et al. may be the primary reason for the discrepancies between the two studies. We developed the Wildfire Atlas of China (WFAC), a robust ground-truthed fire occurrence dataset, in which fire occurrences were detected by multiple satellites (including MODIS) and carefully validated through surface observations by local forestry departments^1^. Even though MODIS-derived fire products, such as active fires and area burned, have much improved in recent years^2^, biases still remain in these products because of surface topography, fast vegetation growth rates, and clouds^2^. In addition to these biases, crop data gaps due to clouds occur in these fire products^2^ and this is especially the case in cloudy subtropical China. As a result, fire patterns in southwestern China have been found to be inconsistent between MODIS fire products and ground observations^3^. To address these biases, we calculated fire occurrences from MODIS fire points^4^ and further distinguished between wildfire and crop fire in the fire occurrence dataset. We find a high fraction of crop fires in the MODIS fire data in northern China, particularly in the North and Northeast China Plains (Fig. 1). These northern Plains are mostly covered by agricultural land rather than forests and routine agricultural crop straw burning accounts for most of the MODIS observed fires in these agricultural areas^5, 6^. Therefore, most fires in northern China detected in the MODIS-derived area burned dataset used by Resco de Dios et al. are crop fires, leading to an overestimation of wildfire activity outside of subtropical China. In addition to this, Resco de Dios et al. calculated the area burned over only the area south of ~35 ^○^N in eastern China, which is only a fraction of subtropical China. Their suggestion that subtropical fires only account for a low fraction of fires in China is therefore geographically biased.

## Spatial patterns of fire occurrences in China

**Fig. 2:**
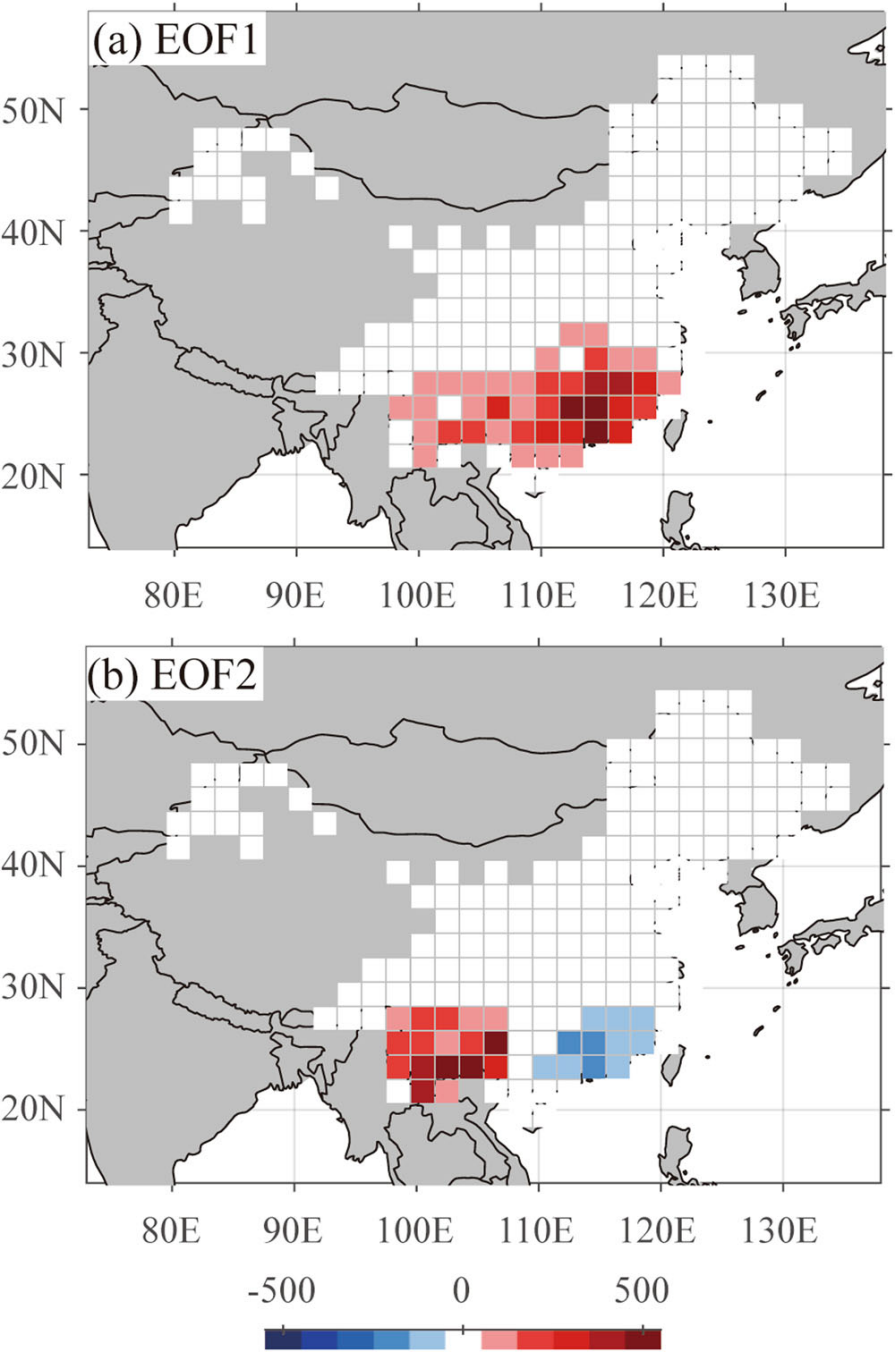
Spatial patterns of the fire occurrences in China. The **a** first and **b** second empirical orthogonal basis functions (EOF1 and EOF2) represent the spatial distribution of wildfire occurrence from the Wildfire Atlas of China (WFAC). These basis functions account for 54 and 19% of total variance.

Based on their analysis of the annual area burned, Resco de Dios et al. argue the lack of a dipole pattern in fire activity between western and eastern subtropical China suggested in our study. We detected this pattern based on the co-varying patterns between the coupled fields of fires in WFAC and sea surface temperature^1^. To support our previous findings, we have now implemented empirical orthogonal function (EOF) analyses on the WFAC field only (Fig. 2). The first and second EOFs represent a monopole and a dipole pattern in subtropical China, accounting for 54 and 19% of the total variance, respectively. Our additional analysis thus confirms the relevance of the dipole pattern of fire occurrences over subtropical China.

## Strong modulation of ENSO on large-scale wildfire activity in China

Resco de Dios et al. claim that the modulation of ENSO on fire in China is weak. They base their claim on the insignificant correlations they find between gridded area and ENSO indices on individual grid points in China. Unlike their analysis of individual grid points, our analyses were based on the covariance of data on these grid points. Combining all grid points, our correlation analysis increases the degree of freedom, raises the likelihood of a significance test, and therefore is reliable and robust. Fire in individual grid points can be noisy on a local scale, while climate plays a more critical role in modulating large-scale fires.

Many previous studies revealed the dominant impacts of ENSO in different regions of China^7, 8^. Resco de Dios et al. stated that the ENSO could only influence the ignitions and thus has little effect on fire activity. In fact, fuel availability and flammability are also key factors in fire occurrence, particularly for large-scale fires^9^. This is evidenced by the strong correlations between fire occurrence and interannual climate variability.

China’s fire policy not only suppresses existing fires but also prevents human-ignited fire occurrences. As revealed in previous studies, the fire suppression policy since 1987 decreased not only burnt areas but also fire occurrences^10^.

The study by Resco de Dios et al. was based on MODIS-derived annual area burned, which differs from our ground-truthed WFAC fire occurrence dataset. The MODIS cannot sufficiently distinguish the wildfire from the frequent crop fires and thus vastly misinterrupt the crop fires as wildfire, especially over the northern plains where forests are rare. Here, we show that the EOF analyses of the WFAC can also reveal the dipole fire pattern between southwestern and southeastern China. We highlight that the dipole fire pattern and ENSO modulation are on large scales. The fire control policy not only suppresses existing fires but also prevents human-ignited fire occurrences, and thus plays an effective role in reducing five activities in China.

## Data Availability

The Moderate Resolution Imaging Spectroradiometer (MODIS) fire datasets (MOD14A1 and MYD14A1 products) are public available at the National Aeronautics and Space Administration (NASA; https://modis.gsfc.nasa.gov/data/dataprod/mod14.php). The Wildfire Atlas of China (WFAC) is available from the supplementary materials of a paper published in Nature Communications (https://www.nature.com/articles/s41467-021-21988-6#Sec10).

## Code availability

The processing codes used in this study are available from the corresponding authors upon request.
